# Addressing Financial Barriers to Health Care Among People Who are Low-Income and Insured in New York City, 2014–2017

**DOI:** 10.1007/s10900-022-01173-6

**Published:** 2022-12-03

**Authors:** Taylor L. Frazier, Priscilla M. Lopez, Nadia Islam, Amber Wilson, Katherine Earle, Nerisusan Duliepre, Lynna Zhong, Stefanie Bendik, Elizabeth Drackett, Noel Manyindo, Lois Seidl, Lorna E. Thorpe

**Affiliations:** 1 Department of Population Health, NYU Grossman School of Medicine, 180 Madison Avenue, New York, NY 10016 USA; 2 Health Initiatives Department, Community Service Society of New York, New York, NY USA; 3 New York University-City University of New York Prevention Research Center, New York University Langone Health, New York, NY USA; 4 Bureau of Harlem Neighborhood Health, Center for Health Equity and Community Wellness, NYC Department of Health and Mental Hygiene, New York, NY USA

**Keywords:** Financial distress, Health insurance, Social determinants of health, Healthcare costs, Community health worker, Health care, Financial barriers, Health disparities, New York city, Policy

## Abstract

While health care-associated financial burdens among uninsured individuals are well described, few studies have systematically characterized the array of financial and logistical complications faced by insured individuals with low household incomes. In this mixed methods paper, we conducted 6 focus groups with a total of 55 residents and analyzed programmatic administrative records to characterize the specific financial and logistic barriers faced by residents living in public housing in East and Central Harlem, New York City (NYC). Participants included individuals who enrolled in a municipal community health worker (CHW) program designed to close equity gaps in health and social outcomes. Dedicated health advocates (HAs) were explicitly paired with CHWs to provide health insurance and health care navigational assistance. We describe the needs of 150 residents with reported financial barriers to care, as well as the navigational and advocacy strategies taken by HAs to address them. Finally, we outline state-level policy recommendations to help ameliorate the problems experienced by participants. The model of paired CHW–HAs may be helpful in addressing financial barriers for insured populations with low household income and reducing health disparities in other communities.

**Supplementary Information:**

The online version contains supplementary material available at 10.1007/s10900-022-01173-6.

## Introduction

Largely due to economic exploitation and trauma inflicted systematically over centuries in the United States,[1] Black people and other people of color have been prevented from accumulating wealth and entering higher-income occupations in the same ways as white Americans. These inequitable systems persist in the form of social, economic, and cultural barriers to accessing health and social services.[2–5] The health care delivery and financing systems are particularly suboptimal, even punitive, for those with low household incomes.[6] While some research has assessed financial barriers for uninsured individuals with low household incomes,[7–9] financial barriers to health care among insured people with low household incomes, and the strategies to ameliorate them, have not been as well characterized. A limited number of studies have shown that insured families ineligible for Medicaid but still considered to have low household incomes struggle disproportionately with unaffordable co-pays, deductibles, and prescription drug costs.[10–22] However, it is often the case that communities where a high proportion of households live below the federal poverty level (herein, “low-income communities”), and in which a majority of members have Medicaid or other health insurance, have limited access to information about how to obtain free or discounted medical care and other support to help with out-of-pocket medical expenses.[23] These factors contribute to underutilization of medical care and unmet health needs, potentially leading to lower quality of life, stress, depression, and an increased likelihood of emergency department visits and hospitalization.[10–22].

This article describes financial barriers to health care experienced by public housing residents with low household incomes and health insurance in New York City (NYC), using data from participants enrolled in Harlem Health Advocacy Partners (HHAP).[24] Established by the NYC Department of Health and Mental Hygiene (herein, “NYC Health Department”) in 2014, HHAP is a place-based community health worker (CHW) and health advocate (HA) initiative that aims to close equity gaps in health and social outcomes between NYCHA residents in East and Central Harlem and other New Yorkers through health coaching, health navigation, group wellness activities, increased community awareness, and advocacy.[24] Trained HAs were paired with CHWs to provide health care navigational assistance to help residents find, understand, and use affordable/low-cost health insurance and health care. CHWs addressed specific health needs, supported health goal-setting and behavior changes, and addressed social determinants of health. In this paper, we aim to: (1) characterize self-reported financial barriers to health care experienced by insured public housing residents, (2) describe strategies HAs used to assist clients in overcoming financial and related barriers, and (3) highlight broader strategies and policy approaches for ameliorating common financial barriers to accessing health care services for insured individuals with low household incomes.

## Methods

### Intervention

Led by the NYC Health Department, HHAP was developed as a multi-stakeholder partnership with the New York University-City University of New York Prevention Research Center (NYU-CUNY PRC), the Community Service Society of New York (CSS), and the New York City Housing Authority (NYCHA).[24] HHAP is guided by a health equity framework and the evidence supporting the use of community-based CHWs to improve health outcomes.[4,25–28] HHAP CHWs engage residents with asthma, diabetes, hypertension, and other health conditions, to improve disease management and achieve health goals. CHWs provide health coaching activities, community events, and advocacy training.[24] HHAP strives to hire CHWs who live in public housing and/or the East and Central Harlem neighborhoods to create authentic connections to the community served and a workforce development pipeline through full-time employment.

**Fig. 1:**
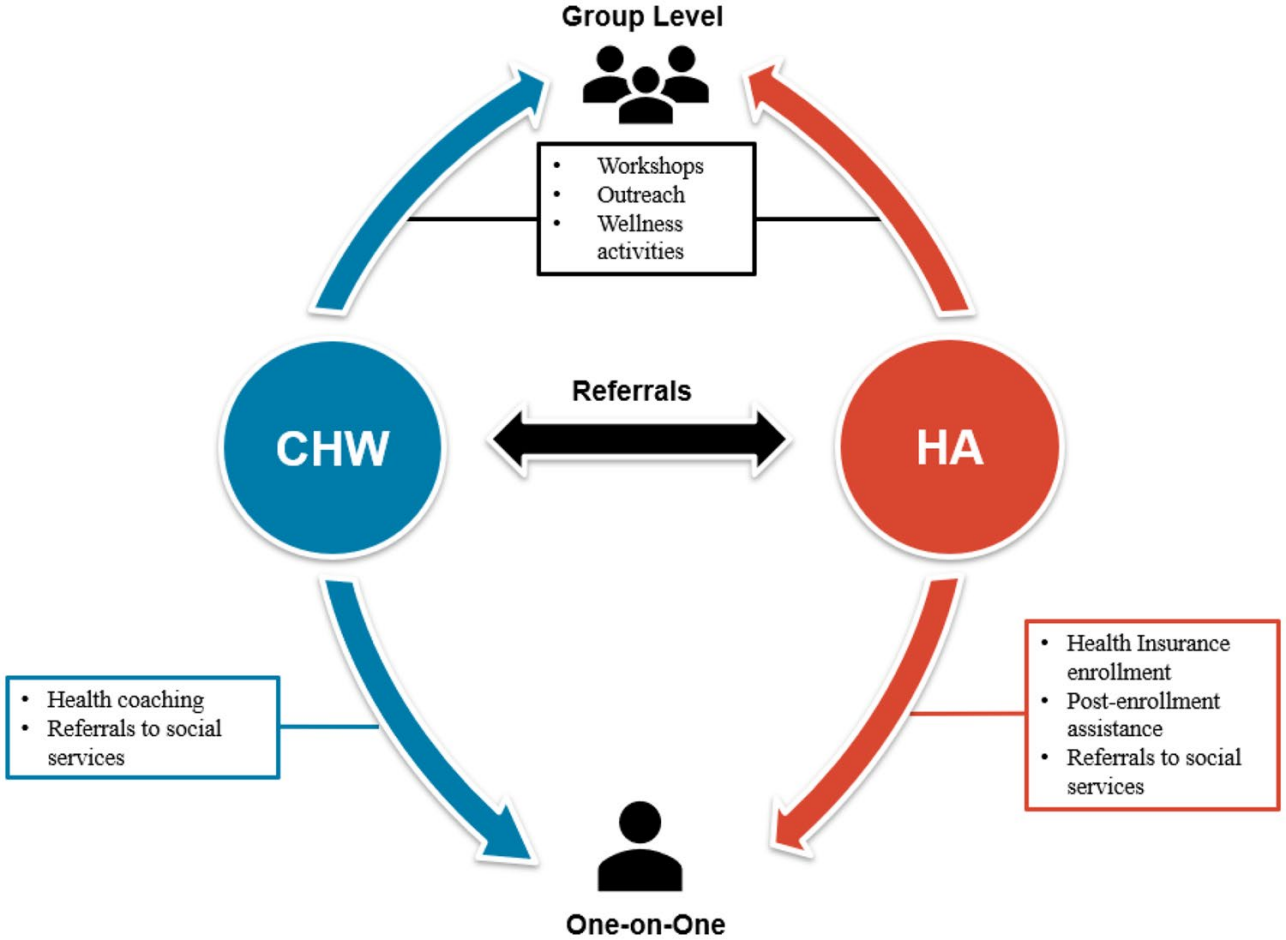
Model of Integrated Services Provided to New York City Public Housing Residents through the Harlem Health Advocacy Partners (HHAP) Initiative

A novel feature of the HHAP model combines CHW assistance with that of HAs, who have technical expertise in health insurance and health system navigation.[24] This component was conceived in response to feedback from the community and evidence in the published literature that many insured residents in low-income communities face health care navigation challenges.[29,30] The HA component allows CHWs to refer residents to HAs when there are insurance-related obstacles to achieving their health goals. HAs use their knowledge of both public and private insurance systems and the local health care provision landscape to help residents understand their insurance options, obtain affordable health care, resolve billing issues, and overcome other barriers to getting the health care they need (Fig. 1).[24] CHWs work one-on-one with residents to achieve their health goals and HAs use their specialized training in health system navigation and insurance policy to mitigate barriers to achieving those goals.[24].

At HHAP’s inception, CSS was contracted to provide HA services to the program. The history of CSS’s Health Advocates can be traced back to 1998 when the NYC Council provided funding to CSS to establish the Managed Care Consumer Assistance Program in partnership with 26 community-based organizations (CBOs). This program was New York’s first health care Consumer Assistance Program (CAP) and a precursor to federal CAPs, which were authorized in Sect. 1002 of the Patient Protection and Affordable Care Act. CAPs educate consumers about coverage options, enroll consumers into coverage, inform consumers of their rights and responsibilities, file grievances with insurance plans and regulators, and report back to policy makers about problems with existing programs and regulations. CSS has operated New York State’s (NYS’s) CAP, Community Health Advocates (CHA), since 2010. CHA, currently funded by the NYS State Legislature and the NYS Department of Health, is one of the largest CAPs in the country with a live-answer Helpline and a network of 27 CBOs. The inclusion of HA services provides assistance to clients whose health goals are hampered by financial barriers related to their health care and coverage, particularly in light of the subsequent reductions in federal funding for state-based CAPs, which began in 2017.[31].

**Fig. 2:**
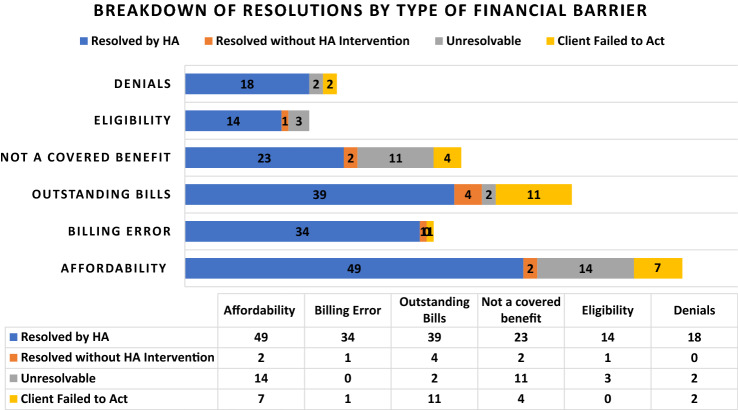
Breakdown of Resolutions by Type of Financial Barrier

Because HHAP is place-based, it eliminates the burden of traveling to receive health insurance enrollment or other post-enrollment assistance for clients. HHAP participants can meet with an HA in their homes, a community center, or neighborhood health action center. Unlike other place-based initiatives that train CHWs to identify a client’s issue and refer them to other services for resolution, HAs focus on sustaining relationships with CHWs and clients through advocacy and addressing barriers to health care, while also expanding health insurance literacy.[32–37] Specifically, HAs help residents understand their insurance options, obtain affordable health care, resolve billing issues, and overcome other barriers to getting needed health services (Fig. 2).[24] Some insurance navigation issues can be addressed in a single visit. Other issues require multiple conversations with residents and additional action by HAs.

### Data Sources

Data for this analysis were drawn from two data sources used to conduct formative and program evaluations for HHAP: focus groups done in the planning phase of HHAP and administrative data record review from the first four years of HHAP. At the outset of the project, the NYU-CUNY PRC team conducted focus groups in December 2014 among HHAP-eligible NYCHA residents.[38] Focus group participants were required to meet the following criteria: (1) speak English or Spanish; (2) be aged 35 to 65 years; (3) have a self-reported diagnosis or family history of diabetes, asthma, and/or hypertension; and (4) reside in one of five intervention housing developments. Focus groups lasted approximately 1.5 to 2 h; six focus groups were completed with a total of 55 residents.[38] Discussion topics included barriers and facilitators to disease management, engaging in healthy behaviors, preferred methods of health education and promotion, and acceptability of and motivation to participate in a CHW intervention.[38].

The second data source focused specifically on HHAP participants who were referred to CSS for HA assistance. We reviewed CSS administrative case record notes and compiled case studies from September 1, 2014 through September 30, 2017 to provide further context for problems faced by CSS clients reporting financial barriers to care and the process by which HAs resolved these problems. Within this timeframe, HHAP HAs assisted 591 public housing clients, with 150 individuals (25%) who had insurance reporting financial barriers to care. We identified 244 financial barriers among the 150 clients, with clients having the option to report multiple barriers.

### Data Analysis

Recorded focus group sessions were transcribed, and a codebook was developed using the moderator guide as an initial outline of primary codes, followed by secondary and tertiary codes. Three independent coders reviewed and analyzed transcripts through thematic analysis, following a constant comparative approach, discussing and resolving discrepancies until an acceptable level of inter-coder reliability had been established.[39] Atlas.ti 6 was used to code and analyze the data.[39,40] Findings related to barriers and facilitators to access to care were highlighted for this paper.

Data analysis from the CSS administrative record review was conducted on client cases reporting financial barriers to care. Each CSS client case was coded into one of six categories of financial barriers: (1) Affordability; (2) Outstanding Bills; (3) Non-covered Benefits; (4) Billing Errors; (5) Service Denials; and (6) Eligibility. These codes were created based on major themes presented in the data. *Affordability* barriers involve cost-sharing, such as deductibles, co-insurance, co-pays, or premiums that prohibit individuals from accessing care. *Outstanding Bills* include medical bills indicating a balance that clients are responsible for paying. *Non-Covered Benefits* include services not covered by insurance and cases in which plan benefits were exhausted. *Billing Errors* include bills sent to clients erroneously, either due to health insurance system or provider error. *Service Denials* include cases in which clients sought to appeal or otherwise contest pre-authorization or service coverage denials from their health insurer. *Eligibility* includes the inability to enroll into supplemental health insurance.

Resolutions for each financial barrier were coded into one of four categories: (1) Resolved with HA assistance, indicating that an HA directly resolved the issue or provided information towards resolution); (2) Resolved without HA assistance, indicating cases resolved by the client or an administrative body without HA advocacy; (3) Unresolved due to issues outside the scope of HAs, such as an unmet eligibility requirement or a plan contract; and (4) Unresolved due to loss to follow up. HAs participate in weekly case review sessions, and upon closure, cases undergo a quality assurance process to ensure all options available to address the client’s needs were explored.

Descriptive analysis was conducted of the codes generated from the CSS database to characterize the proportion and type of barrier experienced by clients as well as the proportion and type of resolutions. In addition to this descriptive analysis, we conducted a content analysis of CSS case files to generate case studies of financial barriers to care and resolution processes and strategies.

## Results

### Characterization of Barriers to Health Care Among East and Central Harlem NYCHA Residents

During focus groups to explore barriers and facilitators to disease management, residents were asked questions about care management and financial barriers to care. Participants were mostly female (82%), with an average age of 58 years. Participants described affordability issues related to their insurance coverage as a significant barrier to chronic disease management and quality of life.

*With me, it seems like my copayments are going up higher and higher even though I am taking the same pill. The medicine is just costing more. I don’t know if [it’s] like the insurance I have*.

*You just have to accept it because that is the policy. I feel like each one has a[n] “I am going to get you” part in it. I worked my entire life and then some, and now I have to pay a phenomenal amount of money just because I went to the hospital….*

Many participants with low or fixed incomes cited outstanding bills and difficulty covering premiums, co-pays, and other out-of-pocket expenses.

*Sometimes the [insurance] doesn’t cover the diabetic medication. So, some people can’t get the medication because they are on a fixed income.*

Participants also expressed frustration with navigating health coverage policies and lack of coordination or consistency among providers, insurers, and pharmacies.

*I had a doctor that I had for 10 years and he knew me, but when they started this HMO thing, I keep getting a new doctor every 6 months. I don’t get a chance to know my doctor.*

*It’s trying to find the right doctor that can use your insurance. I continue to move until I find someone that I am comfortable [with]. I’ve changed so many times; I’m just used to changing because I want to find one that is comfortable.*

*They don’t give you enough time to know the doctor before he or she is gone.*

Participants were particularly distressed by the financial consequences of this lack of coordination.

*A lot of the insurance - they do not accept things like the cuff. They want me to pay out of pocket, which I can’t. It’s hard because you don’t know what the insurance pay[s] for and what it doesn’t pay for. The doctor says you need this, and you need that, and it is like how am I supposed to get it?*

*If you are hospitalized and you need anesthesia, they are working on a team. And then they need your insurance, but then you find out, this guy [was] working on you that was not covered by the insurance. They need to know that certain doctors are covered. They shouldn’t have had him on the team in the first place.*

*And then sometimes my high blood pressure medicine; they were paying for it. Then they said no and that I should be paying something else….I was out of medicine for 3 weeks. Then they give you approval for just one year and you have to do it again.*

### Financial Barriers to Care Experienced by CSS Clients

Of the 591 HHAP clients served between November 2014 and January 2017, 150 individuals (25%) reported experiencing a financial barrier to care. Although the provision of demographic information was not required to receive HA services, 96% (n = 144) of the 150 individuals who reported experiencing a financial barrier also reported some demographic information. Of those who reported demographic information, 74% (n = 106) were female, 50% (n = 58) identified as Hispanic, and 48% (n = 56) identified as African American (Table 1). 63% (n = 90) and 35% (n = 51) reported speaking English and Spanish at home, respectively. Clients who experienced financial barriers to accessing care (n = 150) were more likely to be African American (48% vs. 33%) or Hispanic (50% vs. 40%) compared to those who did not experience financial barriers (n = 441; see **Supplement**).

**Table 1:** Demographics and insurance type among health advocate clients with financial barriers to care, harlem health advocacy partners (HHAP) initiative, sept. 1, 2014–sept. 30, 2017 (N = 150)

Demographics			Insurance Type					
			Medicaid		Medicare		Other	
	**N**	**%**	**N**	**%**	**N**	**%**	**N**	**%**
Age Range								
18–45	5	5	4	80	0	0	1	20
46–64	52	49	17	33	27	52	8	15
65 or older	49	46	3	6	46	94	0	0
Did not report	44							
Gender								
Female	106	74	28	26	70	66	8	8
Male	38	26	7	18	27	71	4	11
Did not report	6							
Race/Ethnicity								
African American	56	48	13	23	40	71	3	5
Hispanic	58	50	17	29	37	64	4	7
Other	3	3	2	67	1	33	0	0
Did not report	33							
Language at Home								
English	90	63	20	22	60	67	10	11
Spanish	51	35	15	29	34	67	2	4
Other	3	2	0	0	3	100		0
Did not report	6							
Chronic Condition (Self-Reported)								
No	32	22	12	38	15	47	5	16
Yes	112	78	22	20	83	74	7	6
Did not report	6							
Household Income (Self-Reported)								
Less than 15 K	63	56	19	30	41	65	3	5
$15,000-$25,000	36	32	5	14	28	78	3	8
$25,001-$40,000	11	10	0	0	9	82	2	18
$40,001-$60,000	3	3	0	0	1	33	2	67
Did not report	37							
Household Size								
1	58	46	11	19	41	71	6	10
2	44	35	9	20	33	75	2	5
3	11	9	6	55	5	45	0	0
4+	13	10	3	23	6	46	4	31
Did not report	24							

65% (n = 97) of clients who experienced financial barriers were enrolled in Medicare through original Medicare or a Medicare Managed Care plan. Of the Medicare enrollees, 26% (n = 39) were dual-eligible, with Medicaid as their secondary insurance. A total of 23% (n = 34) of clients had Medicaid as their primary insurance (17% enrolled in a Medicaid Managed Care plan and 6% enrolled in fee-for-service Medicaid). 6% of clients (n = 9) had employer-sponsored insurance, 2% (n = 3) were enrolled in the Essential Plan, the NYS Basic Health Program serving lower income and immigrant populations), and 4% (n = 6) did not report insurance type.

Clients who reported financial barriers experienced a total of 244 financial barriers (ranging from one to three barriers per client). Affordability barriers were the most common and were experienced by 48% of the 150 clients with barriers. This was followed by outstanding bills (37%), non-covered benefit barriers (27%), billing errors (24%), service denials (15%), and eligibility issues (12%). A total of 73% of the 244 financial barriers were resolved by HAs, with at least one barrier (clients often experience more than one) resolved for 79% (n = 118) of clients. For the remaining barriers, 13% were unresolved due to issues outside the scope of HAs, 10% were unresolved due to client loss to follow up, and 4% were resolved without HA intervention (Fig. 2).

#### Affordability

Forty-eight percentage of clients experienced affordability barriers, which accounted for 72 of the 244 barriers experienced. Many insured clients had difficulty affording their monthly premiums and cost-sharing responsibilities, such as deductibles and co-pays. These affordability barriers were most often related to Medicare.

HAs were able to resolve 49 of the 72 affordability barriers using various strategies. Premiums and cost-sharing were reduced for some Medicare enrollees through supplemental insurance programs like the Medicare Savings Program (MSP).[41] HAs also assisted residents who were ineligible for supplemental insurance. HAs often directed clients to HHC Options, which provides care on a sliding fee scale through NYC’s public hospital system or through alternative private philanthropy resources, like The New York Times (NYT) Neediest Cases Fund, as demonstrated through the case below.[42–44]

*A 72-year-old client with advanced stage periodontitis needed assistance from an HA to secure coverage for her procedures. The client’s Medicare only covered preventive dental services and her supplemental dental coverage only offered a 20% discount on services. The client is the primary caregiver for her grandchildren and could not afford to pay the remaining costs. The HA helped her find a dentist who would treat her at a lower cost than any standalone dental insurance on the market could offer and helped her secure a NYT Neediest grant of $930 to pay for the procedure.*.

#### Outstanding Bills

Thirty-seven percentage of clients experienced unexpected medical bills, which accounted for 56 of the 244 barriers experienced. Most bills experienced by residents could be categorized as cost-sharing bills, balance bills, hospital out-of-network emergency service bills, surprise bills (unexpected bills for out-of-network care received unbeknownst to patients), bills for non-covered services, bills for services denied on the basis of medical necessity, and bills for failure to request a prior authorization. HAs had to be well-versed in operational and legal intricacies of the health care and insurance systems and apply a multifaceted approach to solving outstanding bill issues. Because of the fragmented nature of health care coverage in the U.S., the HA’s decision on which strategy to use to achieve a positive outcome depended not only on the type of bill but also on the resident’s insurance type. An example of strategies used to resolve unexpected medical bills experienced by a client is described below.

*A client had over $14,000 in outstanding medical bills. The client had not worked since 2014 due to a work-related injury and had a pending workers’ compensation case. The coverage from the client’s former employer was still active, but the plan had unaffordable co-pays and a high deductible. The HA helped the client apply for Medicaid as secondary insurance through the NYS of Health Marketplace and resolved many of the bills through the retroactive approval of coverage.*

In Table 2, we summarized common billing issues experienced by HHAP clients, the strategies employed by HAs to address them, and broader policy recommendations to resolve underlying structural drivers of the recurring billing issues consumers encounter. Medical billing can be challenging for consumers to navigate without an understanding of the specific billing protections for the type of plan in which they are enrolled and that plan’s contract details. Robust consumer assistance programs are needed to ensure that plans adhere to billing protections and provide any mandated coverage to their members. A recent report indicated that “[e]ighteen percent of payment denials were for claims that met Medicare coverage rules and [Medicare Advantage Organization] billing rules.”[45] Several of the issues identified in Table 2 are discussed in turn below.

**Table 2:** Common issues regarding outstanding bills, client strategies, and policy recommendations

Issue	Definition	Advocacy strategies	State-level policy recommendations
Hospital/provider cost-sharing bills	Bills from hospital or provider such as copayments, coinsurance, deductibles, or facility fees	Verify debt or charges against client’s plan contract. If client is unable to pay: • Help client apply for Hospital Financial Assistance (HFA) • Negotiate with hospital/provider (e.g., find fair market price for service(s) performed, write financial hardship letter, offer lesser lump sum payment, and/or set up a payment plan) • Help Medicare-eligible clients enroll in the Medicare Savings Program	Increase price transparency to help patient avoid high-cost providers.
	Medicare requires members to pay 20% coinsurance for most hospital/medical care.		Standardize provider billing practices and issue only one bill for hospitalizations and procedures
	Clients with commercial insurance have higher cost-sharing than individuals enrolled into public insurance programs like Medicaid, Essential Plan and Child Health Plus		Prohibit providers from holding patients accountable for facility fees unrelated to medical services. Such fees should be negotiated between providers and insurers
Hospital/provider balance bills	Balance billing is when a patient receives a bill for the difference between the amount paid by the health plan and the amount charged for services	Verify hospital/provider has accurate insurance information. Determine if balance billing protections apply, such as: • Members of Medicaid and Qualified Medicaid Beneficiaries in a Medicare Savings program are protected against balance billing. • Hospitals that are part of an HMO network cannot balance bill commercial, fully-insured enrollees. • HAs can help negotiate balance bills if necessary (see above)	Expand balance billing protections to protect patients who receive erroneous information from their provider or plan regarding in-network providers
Hospital out-of-network emergency services bills	Emergency Room bills from out-of-network (OON) providers in an in-network or OON hospital	• If client has a fully-insured commercial plan, they should be held harmless. If the plan does not comply, HA can help the client file a complaint with Department of Financial Services (DFS) • If client has a self-insured commercial plan verify if No Surprises Act protections apply. If not, help client participate in an Independent Dispute Resolution (IDR) • Help clients apply for hospital financial assistance or to negotiate down their medical bills	Prohibit OON billing for emergency services, defined to include all hospital, physician and ambulance charges, and any other pre-emergency services.
			Create an independent dispute resolution process for plans and providers, and prohibit all balance billing for emergency services
Surprise bills	A bill is a surprise bill if: • Bill is from an OON provider at an in-network hospital and either: • in-network doctor was not available; • client did not know an in-network physician provided services; or • unforeseen medical circumstance arose at time services were provided • A client is referred by an in-network provider to an OON provider and client is not aware the provider they were referred to was OON. Only applicable if plan requires referrals	If client has a fully-insured commercial plan, HAs help the client complete and submit an assignment of benefits (AOB) form to be held harmless for the bill	Extend balance billing protections to patients who receive false information about a provider’s inclusion in a network from either the provider or the plan.
		If the plan does not honor the AOB or mistakenly process the AOB as an appeal, file a complaint with DFS	Apply these protections in all instances when patients unknowingly receive care from an out-of-network provider
		If client has a self-insured commercial plan or is uninsured, use same approach as Hospital out-of-network emergency services bills	
Hospital/provider bills for non-covered services	All health insurance plans can deny coverage and bill for a service on the grounds that it is “not a covered benefit”, meaning that the service is excluded from the plan’s contract	Verify that the plan is not violating any applicable federal or state laws by failing to cover the service.	Expand coverage for preventative care services to include ultrasounds in lieu of mammograms for women with dense breast tissue.
		Review the plan contract. If the plan is not responsible to cover the service, help client apply for hospital financial assistance or negotiate the bill	Expand coverage of IVF and fertility services and improve dental coverage and benefits
Hospital/provider bills for services denied on medical judgement	Both private and public health insurance plans can deny coverage for services deemed not meeting the plan’s clinical criteria, or if the service is from an OON provider and not materially different from the in-network service	The HA can help the client file an internal and/or external appeal • Fully-insured – Department of Financial Services (DFS) external appeal • Self-insured – Independent Review Organization (IRO) external appeal	Provide robust consumer assistance programs and include contact information for programs on all claim denials
		If the appeal is lost, help the client apply for hospital financial assistance or negotiate the bill	Maintain external appeals databases so that consumer and advocates can review past decisions
Hospital/provider bills for services that need prior authorization	Many insurance plans require prior authorization before they will cover certain medical services or medications. Clients who fail to request prior authorization may be billed for services	Verify the medical service requires prior authorization. A client may be held harmless when an in-network provider fails to obtain prior authorization	Limit the types of care that require pre-authorization. For example, New York State recently prohibited prior authorization for pediatric mental health hospitalizations
		Request that the provider submit prior authorization retroactively. If that is unsuccessful, help the client apply for hospital financial assistance or negotiate the bill	States must provide independent consumer assistance programs to aid patients in navigating prior authorization requirements for care

#### Non-Covered Benefits

Twenty seven percentage of clients experienced non-covered benefits, accounting for 40 of the 244 barriers. HAs resolved 58% of these barriers. The HAs educated clients about their rights when benefits were exhausted and helped them explore secondary and tertiary insurance options. For instance, HAs helped clients enroll in the NYS Elderly Pharmaceutical Insurance Coverage (EPIC) program, a supplemental insurance option for NYS residents who are 65 and older, enrolled in Medicare Part D drug coverage, and are unable to afford the cost of a necessary prescription medication or are caught in the annual Medicare “doughnut hole.”[46]

*A client questioned why several of her medications were not covered by her insurance. Each prescription was either a Tier 2 or 3 drug, for a total monthly cost of $145. None of the prescriptions had cheaper generic alternatives, which the client preferred. The HA introduced the client to the EPIC program as an option. In EPIC, members may pay an annual fee ranging from $8 to $300 based on their income. After any Part D deductible is met, members then only pay the EPIC co-payment for drugs. Co-payments range from $3 to $20 based on the drug cost not covered by Part D. This client was eligible to pay $230 annually or $52.50 quarterly, allowing her to receive her prescriptions at a more affordable rate.*.

#### Billing Errors

Billing errors were common among clients enrolled in both Medicare and Medicaid. They were experienced by 24% of the clients in our sample and accounted for 36 of the 244 barriers reported. HAs resolved 94% of these barriers, often by advocating with billing offices to correct errors by citing the Medicaid law that protects dual-eligible clients from balance billing and ensuring that claims are properly resubmitted. Billing errors also occurred when a client with a Medicaid spend-down was hospitalized; Medicaid limits the amount that hospitals can charge these patients. HAs advocated to ensure that clients were only held responsible for the allowable amount and negotiated that amount down further based on the client’s ability to pay.

HHAP clients with Medicaid coverage often had billing errors related to out-of-state emergency medical care. Medicaid covers emergency care in all 50 states, but it can be difficult to get out-of-state providers to honor this provision. Through advocacy, HAs helped Medicaid clients resolve these out-of-state emergency bills.

HAs also advocated on behalf of clients confronted with out-of-network surprise bills by using their knowledge of recently passed consumer protection laws, like NYS’s 2015 Surprise Bill Law. Trained to navigate complex and unique health insurance issues, HAs held billing offices accountable, while also educating clients about their rights, as demonstrated by the case described below.

*A client received a denial from his health insurance company claiming he had gone to an out-of-network provider for cataract surgery and would need to pay the out-of-network rate of $3,500. However, this provider was listed on his Explanation of Benefits as being in-network. The client worked with the HA to inquire into this discrepancy and request that the health insurance company review the determination, before filing an appeal of the decision. The insurance company resubmitted the claim, and the bill was ultimately covered as in-network.*

#### Service Denials

Service denials for medically necessary treatments and procedures were experienced by 15% of clients in our sample and accounted for 22 of the 244 barriers. These denials can significantly delay or derail medical care when a client does not have the technical expertise or advocacy support to appeal. HAs resolved 81% of these denials. To resolve these types of issues, HAs identified errors in billing codes, submitted appeals in disputes over medically necessary procedures/services, and discovered administrative errors or unnecessary delays. Through appropriate channels and advocacy, HAs helped their clients obtain the care they needed, as demonstrated by the cases below.

*A client needed a special prosthetic for a knee surgery because of an ongoing infection. The client’s insurance refused to cover the alternative prosthetic. The HA investigated the case and determined that the provider had used an incorrect billing code. The HA helped the provider resubmit the authorization with the correct information and the client was able to get her surgery.*

*A client’s 14-year-old son was denied braces by his Medicaid plan. The child was in a lot of pain and having trouble concentrating at school. The HA marshalled medical evidence from an orthodontist to support the client’s appeal of the insurance denial but lost. The HA subsequently helped the family secure a NYT Neediest Cases grant for $3,004, and the child was able to get his braces.*

#### Eligibility

12% of clients experienced eligibility barriers, which accounted for 18 of the 244 barriers experienced. HAs resolved 78% of these barriers. Some HHAP clients were eligible for but unable to enroll in health insurance that would cover necessary medical care. HAs helped clients overcome barriers to enrolling in coverage. Examples include clients who delayed Medicare enrollment and faced a late penalty, and clients who had Medicaid coverage with a surplus and thus were unable to enroll into a managed long-term care plan to cover homecare services. An example of an eligibility barrier and resolution that an HA provided is described further below:

*A client became eligible for Medicare after 24 months on disability, but she was unable to enroll into Medicare Part A because she was mistakenly being asked to pay a monthly premium of over $400. As an SSI recipient, the client’s eligibility to enroll should not have been contingent upon her ability to pay this premium. Through advocacy, her HA was able to remove this obstacle and enroll the client into Part A without monthly premiums.*

## Discussion

In this paper, we contribute to a limited body of literature to gain a more in-depth understanding of health care-associated financial barriers faced by insured individuals with low household incomes using qualitative and administrative data.[4,17,47] We also describe an innovative, place-based model to ameliorate these barriers. Focus group data revealed residents to be deeply frustrated with the lack of coordination in the health care system and often unable to afford cost-sharing for health care services.[38] Our analysis of administrative data identified several categories of financial barriers faced by HHAP clients in accessing care and outlined strategies HAs used to resolve the majority of the most frequently reported financial barriers to health care. Clients whose issues could not be resolved were informed of the reasons why their barrier could not be addressed. HAs educate patients about both their rights and the limitations of those rights in the health care system, a key component of health care literacy.

In the HHAP initiative, HAs employed direct navigation and advocacy methods while also addressing health care literacy, rather than using passive referral methods.[48–50] HAs must be highly trained on the complexities of the health care system and its programs, as well as resources and tools to resolve clients’ financial barriers. Their core strategies included helping residents apply for hospital financial assistance or supplemental insurance programs, enforcing consumer protection laws, and appealing service denials. For certain financial barriers, such as outstanding medical bills, HAs had to resort to a wider arsenal of advocacy tools to help clients eliminate or reduce the amount they owed.

HA decisions on which advocacy tool to use and outcomes of HA intervention depended heavily on the resident’s insurance type. This was common across nearly all financial barriers cases and highlights one of the major challenges faced by broad-based health care literacy initiatives. For instance, residents enrolled in public programs like Medicaid, Child Health Plus, and the Essential Plan were less likely to face affordability barriers and cost-sharing bills because these types of insurance do not charge deductibles or high co-payments for services. In general, Medicaid enrollees were more protected from financial barriers such as balance billing than residents enrolled in Medicare or commercial health insurance products because providers are not allowed to charge a difference between the amount paid by a Medicaid plan and the amount charged by the provider.[10–22] Lack of financial consumer protections and affordable cost-sharing options were the main reasons residents enrolled in Medicare reported more financial barriers. HAs thus tried to help senior residents reduce their Medicare premiums and cost-sharing by enrolling them in Medicaid or the Medicare Saving Program. However, income limits for some of these programs are so low that even clients at 101% of the Federal Poverty Level were not eligible to enroll.

While this study demonstrates the valuable role that HAs can play in ameliorating financial barriers, some financial barriers remained intractable. Medicare enrollees ineligible for supplemental insurance plans and other cost-sharing reductions such as the pharmaceutical discount can have significant out-of-pocket expenses associated with their health care. Several benefits are not covered by Medicare—for example, dental and homecare—and raise additional affordability concerns. Many clients eligible for Medicaid with a surplus are often unable to pay the amount required to activate their Medicaid coverage. Facility fees are another health care cost imposed by providers, but sometimes not covered by insurance carriers, resulting in unexpected and unresolvable costs for consumers. Additionally, generations of economic injustice have created and perpetuated a cycle of financial disadvantage that underpins many poor health outcomes. These are reproduced and reinforced by interconnected discriminatory policies and systems of power. HAs advocate for their clients within the confines of the existing health care infrastructure where gaps in coverage persist in areas such as dental and vision care, homecare, and prescription drugs.[17] High deductible health insurance plans and other significant cost-sharing also prohibit many insured people from accessing care, especially when they are ineligible for affordable supplemental insurance options.[8,17].

This study has some limitations. The administrative data were based on clients’ self-reported financial barriers to health care. Due to the lack of a controlled comparison group, it was not feasible to compare the resolution of financial barriers for clients assisted by HAs to those who did not receive any outside assistance or those who were assisted by other means. However, during our observation period, we observed a modest percentage of resolutions achieved without HA intervention (4% of all identified financial barriers).

## Conclusion

This study describes the financial barriers to health care faced by insured public housing residents in East Harlem, but these barriers are experienced more broadly by Black people and other people of color throughout the United States. We highlight the role that HAs play in ensuring that individuals not only have health insurance, but that they understand and are able to use their health insurance to get the care they need. Based on our experience, we recommend that CHW models incorporate HAs to ensure that health system navigation can be seamlessly integrated and that participants can effectively utilize all aspects of their insurance. In addition to addressing financial barriers, HAs help bridge community-clinical linkages, and reduce health disparities experienced by public housing residents, all of which have implications toward achieving health equity. Experimental or quasi-experimental models to test this partnership model against CHW-only service provision would provide valuable empirical evidence.

## Supplementary Information

Below is the link to the electronic supplementary material.

Supplementary material 1 (DOCX 14.5 kb)

## Data Availability

The data that support the findings of the current study are available from the corresponding author on request.

## Code Availability

Atlas.ti 6 was used to code and analyze the data.
