# Supplementary material for: Addressing Financial Barriers to Health Care Among People Who are Low-Income and Insured in New York City, 2014–2017
Source: J Community Health. 2022 Dec 3;48(2):353–66. doi: 10.1007/s10900-022-01173-6 (PMC10060328; doi:10.1007/s10900-022-01173-6)
Supplement: Supplementary file 1 — Supplementary material 1 (DOCX 14.5 kb) [file 10900_2022_1173_MOESM1_ESM.docx]

| **Supplement. Demographics and Insurance Type among Health Advocate Clients with Financial Barriers to Care versus those with No Financial Barriers (N=591)** | | |
| --- | --- | --- |
|  | **Any Reported Financial Barriers** | **No Reported Financial Barriers** |
|  | **N=150** | **N=441** |
| **Insurance type** |  |  |
| Medicaid | 35 (23%) | 144 (33%) |
| Medicare | 97 (65%) | 131 (30%) |
| Other | 18 (12%) | 166 (38%) |
| **Age group** |  |  |
| 0–18 | 1 (1%) | 27 (6%) |
| 19–45 | 4 (3%) | 84 (19%) |
| 46–64 | 52 (35%) | 126 (29%) |
| 65+ | 49 (33%) | 150 (34%) |
| Did not report | 44 (29%) | 54 (12%) |
| **Gender** |  |  |
| Male | 38 (25%) | 143 (32%) |
| Female | 106 (71%) | 298 (68%) |
| Did not report | 6 (4%) | 0 (0%) |
| **Race** |  |  |
| African American | 56 (37%) | 141 (32%) |
| Other | 3 (2%) | 4 (1%) |
| Did not report | 91 (61%) | 296 (67%) |
| **Ethnicity** |  |  |
| Hispanic | 58 (39%) | 176 (40% |
| Did not report | 92 (61%) | 265 (60%) |
| **Language at home** | |  |
| English | 90 (60%) | 306 (69%) |
| Spanish | 51 (34%) | 129 (29%) |
| Other | 3 (2%) | 5 (1%) |
| Did not report | 6 (4%) | 1 (0%) |
| **HH Income** |  |  |
| Less than 15K | 63 (42%) | 159 (36%) |
| $15K–25K | 36 (24%) | 49 (11%) |
| $25,001–$40K | 11 (7%) | 18 (4%) |
| $40,001–$60K | 3 (2%) | 4 (1%) |
| $60,001–100K | 0 (0%) | 1 (0%) |
| More than $100K | 0 (0%) | 1 (0%) |
| Did not report | 37 (25%) | 209 (47%) |
| **Household size** |  |  |
| 1 | 58 (39%) | 152 (34%) |
| 2 | 44 (29%) | 78 (18%) |
| 3 | 11 (7%) | 37 (8%) |
| 4+ | 13 (13%) | 30 (7%) |
| Did not report | 24 (16%) | 144 (33%) |
